# Anomalous amide proton chemical shifts as signatures of hydrogen bonding to aromatic sidechains

**DOI:** 10.5194/mr-2-765-2021

**Published:** 2021-10-25

**Authors:** Kumaran Baskaran, Colin W. Wilburn, Jonathan R. Wedell, Leonardus M. I. Koharudin, Eldon L. Ulrich, Adam D. Schuyler, Hamid R. Eghbalnia, Angela M. Gronenborn, Jeffrey C. Hoch

**Affiliations:** 1 Department of Molecular Biology and Biophysics, UConn Health, 263 Farmington Ave., Farmington, CT 06030-3305 USA; 2 Department of Structural Biology University of Pittsburgh School of Medicine 3501 Fifth Ave., Pittsburgh, PA 15260 USA

## Abstract

Hydrogen bonding between an amide group and the p- π cloud of an aromatic ring was first identified in a protein in the 1980s. Subsequent surveys of high-resolution X-ray crystal structures found multiple instances, but their preponderance was determined to be infrequent. Hydrogen atoms participating in a hydrogen bond to the p- π cloud of an aromatic ring are expected to experience an upfield chemical shift arising from a shielding ring current shift. We surveyed the Biological Magnetic Resonance Data Bank for amide hydrogens exhibiting unusual shifts as well as corroborating nuclear Overhauser effects between the amide protons and ring protons. We found evidence that Trp residues are more likely to be involved in p- π hydrogen bonds than other aromatic amino acids, whereas His residues are more likely to be involved in in-plane hydrogen bonds, with a ring nitrogen acting as the hydrogen acceptor. The p- π hydrogen bonds may be more abundant than previously believed. The inclusion in NMR structure refinement protocols of shift effects in amide protons from aromatic sidechains, or explicit hydrogen bond restraints between amides and aromatic rings, could improve the local accuracy of sidechain orientations in solution NMR protein structures, but their impact on global accuracy is likely be limited.

## 1 Introduction

In 1988, Levitt and Perutz (1988) identified a putative hydrogen bond between an amino group of asparagine and an aromatic ring of a drug bound to hemoglobin. Similar observations of the π electrons of aromatic rings acting as acceptors for hydrogen bonding have been reported before and since (Klemperer et al., 1954; Mcphail and Sim, 1965; Knee et al., 1987). Later in 1986, Burley and Petsko (1986) surveyed 33 high-resolution protein structures and found further evidence of aromatic hydrogen bonds. Tüchsen and Woodward (1987) subsequently observed an upfield shift in the Gly-37 NH and Asn-44 HN resonances due to a nearby Tyr-35 aromatic group. The measurements from this study allowed Levitt and Perutz​​​​​​​ (Perutz, 1993) to estimate that these interactions contribute around 3 kcal mol ${}^{-1}$ in stabilizing enthalpy, about half as strong as a conventional hydrogen bond. Further evidence of such H bonding came from the 2001 study by Brinkley and Gupta (2001) showing FTIR spectroscopic evidence for hydrogen bonding between alcohols and aromatic rings. The ability of aromatic rings to engage in weakly polar CH– π interactions is well documented, with NMR data from Plevin et al. (2010) in the form of weak scalar ( J ) couplings between methyl groups and atoms in aromatic rings providing direct evidence of these interactions. The study also included a survey of 183 X-ray structures and found 183 putative Me– π interactions. Brandl et al. (2001) surveyed 1154 protein structures from the Protein Data Bank (PDB; wwPDB consortium, 2019) for C–H π H bonds and found 14 087 involving aromatic rings and satisfying their geometric criteria. This is made all the more impressive when considering that Levitt and Perutz (1988) report the partial charges on the C–H group are one-third those on the N–H group (the subject of this paper), suggesting that the interaction studied by Brandl et al. (2001)​​​​​​​ is correspondingly weaker. Another survey of note was performed by Weiss et al. (2001). This complete hydrogen bond analysis of two high-resolution protein structures from PDB found 50 C–H π and two (N,O)–H π bonds.

In addition to their ubiquity, there is some indication of the importance of these interactions. In a 1993 review, Perutz (1993) indicated the potentially wide-ranging importance of these interactions, particularly Armstrong et al.'s (1993) demonstration of their role in stabilizing α -helices. There is also evidence that similar interactions play an important role in protein–ligand complexes (Panigrahi and Desiraju, 2007; Polverini et al., 2008)

Following the example of Tüchsen and Woodward (1987), we seek to use NMR to provide corroborative evidence of aromatic hydrogen bonds. In this paper, we survey the Biological Magnetic Resonance Bank (BMRB) for unusual amide proton chemical shifts and amide–aromatic nuclear Overhauser effects.

**Figure 1:**
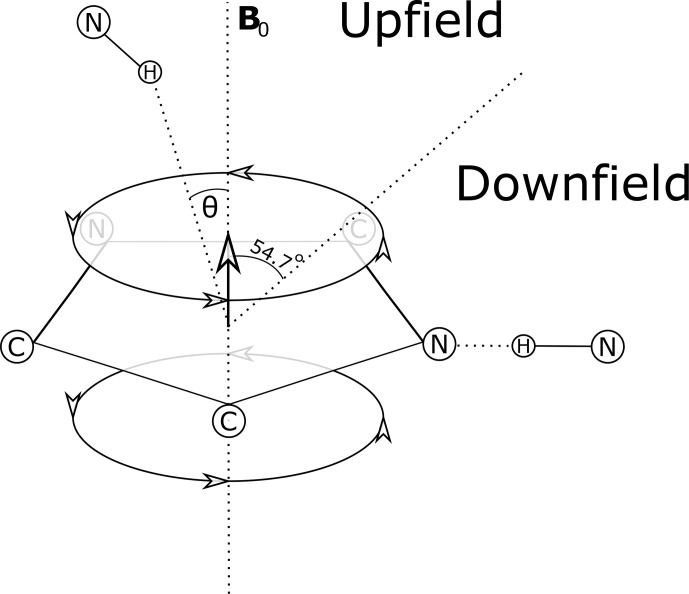
Definition of the azimuthal angle ( θ ) and demarcation of regions of upfield and downfield ring current shifts. For protons above the plane of a Tyr or Phe ring the upfield shift can reach 1.5 ppm for distances from the ring center around 3 Å; for protons in the plane of the ring the downfield shift approaches 2 ppm at 3 Å. For Trp the effects can be significantly larger. Local mobility (e.g. fluctuations about the $\chi_{2}$ sidechain dihedral angle of the aromatic residue) can substantially diminish ring current shifts.​​​​​​​

Theoretical models for the geometrical dependence of the ring current shift include parameterization of quantum-mechanical (Haigh and Mallion, 1979; Memory, 1963) calculations, semi-classical approximation using the Biot–Savart law (Jackson, 1999) for the field arising from current loops (Waugh and Fessenden, 1957; Johnson and Bovey, 1958), and a dipole approximation. For distances from the ring center that are greater than 3 Å above the plane of the ring, and 5 Å in the plane of the ring, the theories all agree well with a dipole approximation (Hoch, 1983). The $(1-3{\mathrm{cos}}^{2}(\theta ))/r^{3}$ geometrical dependence of the field arising from a magnetic dipole (where θ is the angle between the vector from a proton to the aromatic ring center and the vector normal to the plane of the ring) provides vivid explanation for cone separating upfield-shifted from downfield-shifted regions defined by θ=54.7 ${}^{\circ }$ (Fig. 1).

**Figure 2:**
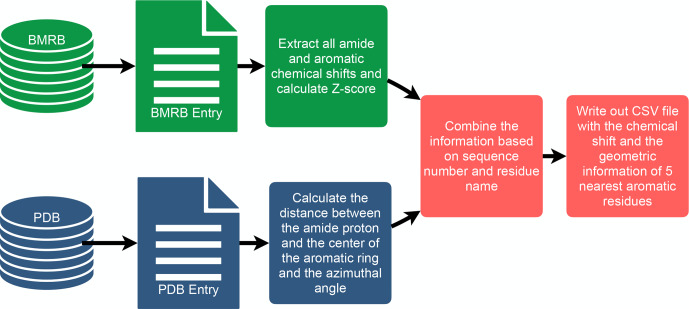
Manual federation of BMRB and PDB via a customized workflow.

**Figure 3:**
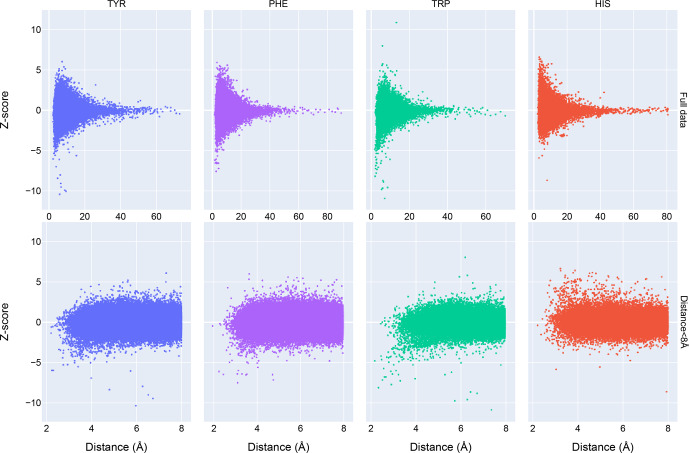
The distribution of amide chemical shifts as a function of the distance of the amide proton from the center of the nearest aromatic ring.

## 2 Approach

To investigate the connection between amide proton chemical shifts and the potential for hydrogen bonding to an aromatic ring, we searched BMRB for assigned amide protons in proteins corresponding to structures deposited in the PDB. BMRB provides the list of BMRB and PDB entry ID pairs via BMRB API (http://api.bmrb.io/v2/mappings/bmrb/pdb?match_type=exact, last access: 15 January 2021​​​​​​​). As of January 2021, we found 7750 BMRB/PDB paired entries and retrieved the BMRB entries (in NMR-STAR format; Ulrich et al., 2019) and PDB entries (in mmCIF format; Bourne et al., 1997) from their respective databases. We filtered out DNA and RNA entries, entries with ligands, oligomers, and protein complexes. In the end we prepared a dataset that consists of 363 686 amide protons from 4670 entries. We combined the chemical shift information from BMRB and the geometric information from PDB for each amide proton and its nearest aromatic ring using sequence number and residue name. For each assigned amide chemical shift, the Z score was computed, characterizing the deviation of the shift from its mean value from the BMRB database

1 $$Z=\frac{\delta_{\mathrm{res}}-{\bar{\delta }}_{\mathrm{res}}}{\sigma_{\mathrm{res}}},$$

where $\delta_{\mathrm{res}}$ is the amide chemical shift of a given residue in parts per million (ppm), and ${\bar{\delta }}_{\mathrm{res}}$ and $\sigma_{\mathrm{res}}$ are the mean and the standard deviation of the amide proton of a given residue type, based on statistics maintained by BMRB (https://bmrb.io/ref_info/stats.php?restype=aa&set=filt, last access: 15 January 2021). For each assigned amide, the distance from the amide position to the center of the nearest aromatic ring is computed from the coordinates in the PDB mmCIF file. The distance is defined as the average of the distance from the amide proton to the center of the aromatic ring, averaged over the members of the structural ensemble present in the PDB entry. For the nearest aromatic ring, we calculated an azimuth angle (Fig. 1), defined as the angle between a vector normal to the aromatic ring plane and the vector between the amide proton and the center of the ring. The ring normal vector is computed by calculating the cross product of two vectors on the plane of the ring (say the vector from the center of the ring to CG and CD1). The table of assigned chemical shifts, Z scores, distances to the nearest aromatic ring, and azimuth angles is provided as a comma-separated text file (CSV file) in the Supplement​​​​​​​. The workflow used in the analysis is depicted in Fig. 2.

**Figure 4:**
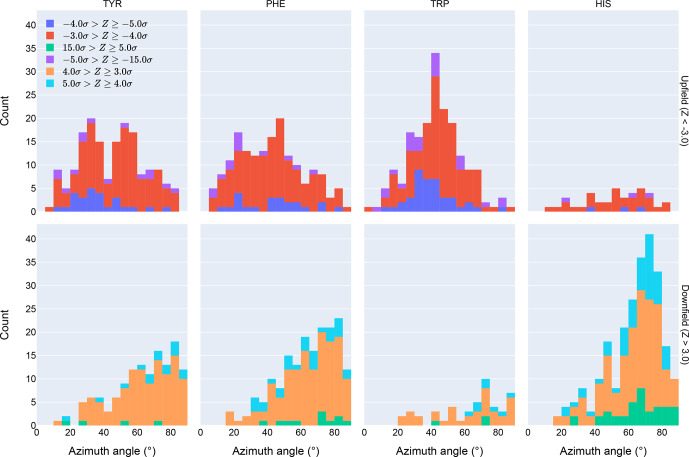
Distribution of azimuth angles for outlier ( >3σ ) amide proton shifts. Upfield shifts are shown in the top row, downfield shifts in the bottom row.

Corroboration of close proximity between an amide proton and an aromatic ring observed in PDB structures is found in assigned distance restraints based on nuclear Overhauser effects (NOEs) present in the BMRB entries. NMR restraint files from the PDB were parsed using PyNMRSTAR (Smelter et al., 2017) for NOE restraints between amide protons and aromatic ring protons of different residues. Because many files list NOEs under “simple” distance restraints, these were included. Due to inconsistencies prevalent in the restraint data, several criteria were implemented to ensure some conformity in the restraints included in our analysis. This and other reasons for excluding entries from the restraints analysis are described in greater detail in Table S1 in the Supplement. Also discarded were individual distance restraints which reported only a lower distance bound or an upper distance bound greater than 6 Å (as this is inconsistent with the nuclear Overhauser effect) and restraints that were ambiguously between more than two different residues (in order to simplify the analysis). Of the entries that remained, 2573 listed at least one restraint between an amide proton and an aromatic ring proton, and 848 did not. For this section of the analysis, the June 2021 ReBoxitory data lake snapshots of BMRB and PDB available on NMRbox (https://nmrbox.org/, last access: 27 August 2021​​​​​​​, /reboxitory/2021/06​​​​​​​) were used.

## 3 Results and discussion

### 3.1 Analysis of chemical shift data

Chemical shift Z scores as a function of distance to the nearest aromatic ring are shown in Fig. 3, separated by the type of aromatic sidechain. For all four aromatic residue types, there is a clear correlation between proximity to the aromatic ring and the amide chemical shift variance: significant deviations from the mean, corresponding to Z scores greater than 2, are most likely when the proton is proximal to an aromatic ring, and the magnitude of the shift deviations are larger for closer proximity. The bottom row in Fig. 3 examines the distribution of amide chemical shifts that are closer than 8 Å in greater detail.

The figure illustrates differences in the pattern of chemical shift deviation for the four different types of aromatic sidechains. For amide protons proximal to Phe, Tyr, or Trp sidechains, there is a noticeable preponderance of upfield shifts (negative Z score). In contrast, His amide protons exhibiting large deviations from the mean tend to be shifted downfield (positive Z scores). The difference in behavior of the outliers for the different aromatic residue types suggests the deviations are not simply the result of residues buried in the protein interior. The upfield-shifted resonances for amides proximal to Phe, Tyr, and Trp are consistent with hydrogen bonding between the amide and the p- π electrons. The downfield-shifted resonances for amides proximal to His are consistent with hydrogen bonding to the electronegative nitrogen atoms of the His ring. In-plane downfield ring current shifts are the same sign as the expected downfield shifts arising from hydrogen bonding, with a predicted amide proton ring current shift of 0.5 ppm for an amide nitrogen distance of 3.4 Å. This is consistent with the observation of larger magnitude Z scores for downfield-shifted amide protons proximal to His.

**Figure 5:**
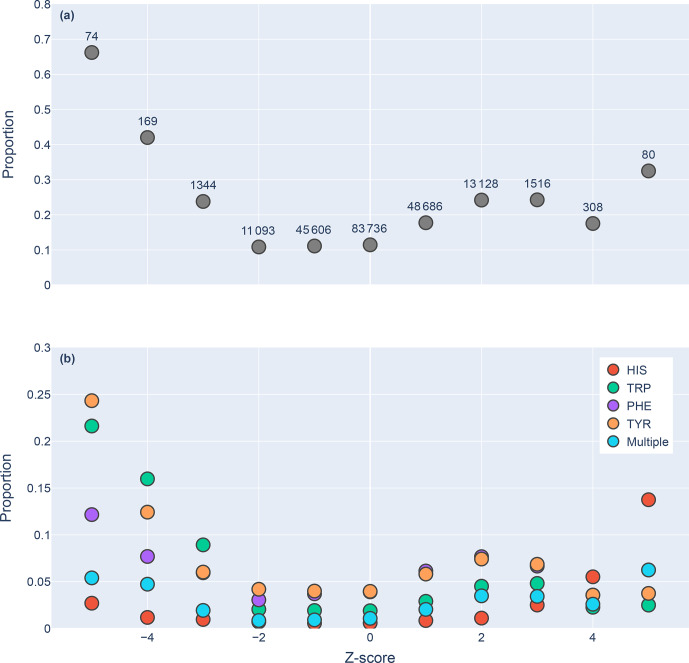
Proportions of amide protons with at least one NOE restraint to an aromatic ring proton ( y axis), as a function of the Z score of the amide proton ( x axis). Proportions are calculated with respect to the total number of amide hydrogens with chemical shifts reported in entries with at least one amide–aromatic restraint. The numbers over each point in panel **(a)** are the total number of such amides (including those lacking any NOE restraints to a nearby aromatic) with that Z score. In panel **(b)**, the restrained amide protons are further demarcated by the type of aromatic sidechain to which they are restrained.

Further evidence of the unusual behavior of amide protons with unusual shifts proximal to His and Trp residues is found in their spatial distribution. Figure 4 shows the distribution of azimuth angles for upfield and downfield outliers that are within 8 Å of an aromatic ring. (Outliers are defined here as having an absolute value of the Z score greater than 3.) Shift outliers proximal to His tend to reside near the ring plane, whereas shift outliers proximal to Trp tend to reside above the ring plane. Phe and Tyr do not exhibit a pronounced preponderance of outliers above or near the ring plane. Interestingly, none of the maxima in the azimuth angle distributions occur at 0 ${}^{\circ }$ , expected for an amide proton directly above the ring centroid, nor 90 ${}^{\circ }$ , expected for an amide proton lying in the ring plane. The peaks near 25 ${}^{\circ }$ observed for Tyr and Phe are close to the value expected for an amide proton 2.4 Å above the ring plane and directly above one of the ring atoms, rather than above the ring centroid.

### 3.2 Analysis of restraint data

We found 31 859 amide protons with at least one NOE restraint to a nearby aromatic ring. Figure 5a shows the proportion of amide protons (from entries with usable restraint data and at least one amide–aromatic restraint) exhibiting these restraints. For both upfield- and downfield-shifted amide protons, the greater the deviation from the mean, the greater the likelihood that corresponding NOE restraints are observed. The trend is noticeably more pronounced for the upfield-shifted amide protons, which is consistent with the formation of hydrogen bonds between the amide and the p- π electrons. The downfield-shifted amides exhibit a weaker correlation, which may be indicative of other dominating effects (not necessarily due to nearby aromatic rings). Figure 5b further demarcates the data by the type of the nearby aromatic residue. We observe the preponderance of amide–aromatic restraints in upfield-shifted amide protons for interactions with Trp and Tyr (and to a lesser extent Phe). In contrast, amide protons proximal to His residues predominate strong downfield shifts ( Z≥4 ). This stands as further evidence for hydrogen bonding from the amide to the p- π electrons in Trp, Tyr, and Phe and to the nitrogen atoms in the His ring.

**Figure 6:**
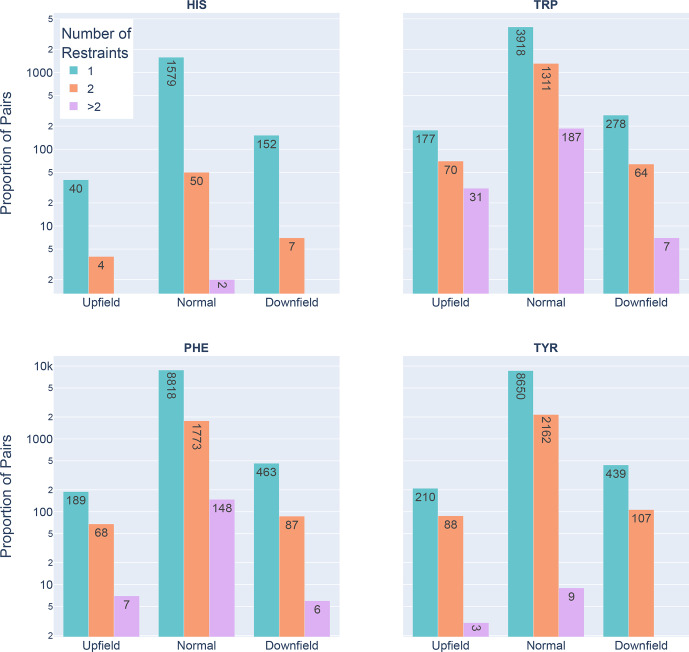
Shown are the number of restrained amide–aromatic pairs (that is amide protons and aromatic rings with at least one defined restraint between them) for the four aromatic residue types and three Z score classifications: upfield ( Z≤-2 ), downfield ( Z≥2 ), and normal ( -2≤Z≤2 ). The colors of the bars correspond to the number of restraints between the pairs; bar heights are plotted using a logarithmic scale.

**Figure 7:**
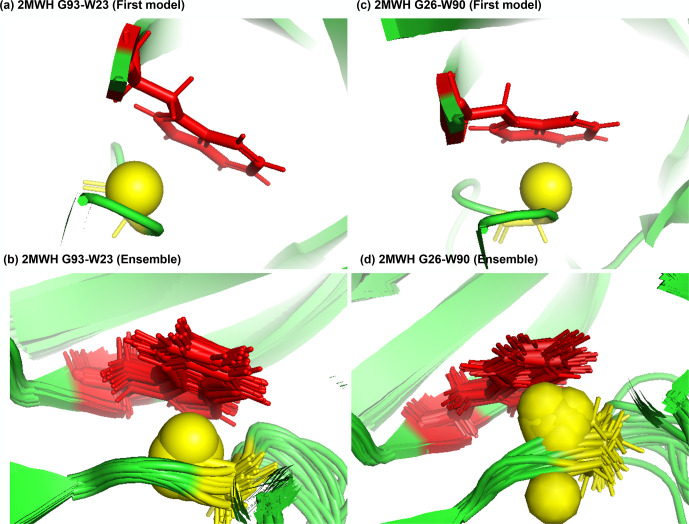
Examples of amide protons with extreme upfield shifts. **(a, b)** PDB:2MWH. The G93 amide proton is directly below the W23 aromatic ring ( Z = - 7, $\delta_{\mathrm{GLY}}$ = 2.937 ppm, d = 3.99 Å, θ = 43.9 ${}^{\circ }$ , ${\bar{\delta }}_{\mathrm{GLY}}$ = 8.237 ppm, $\sigma_{\mathrm{GLY}}$ = 0.770 ppm). **(c, d)** PDB:2MWH. The G26 amide proton is directly below the W90 aromatic ring ( Z = - 6.43, $\delta_{\mathrm{GLY}}$ = 3.38 ppm, d = 2.98 Å, θ = 25.0 ${}^{\circ }$ , ${\bar{\delta }}_{\mathrm{GLY}}$ = 8.327 ppm, $\sigma_{\mathrm{GLY}}$ = 0.770 ppm). The amide proton is represented as a yellow sphere, and the aromatic sidechain is shown in red.

**Figure 8:**
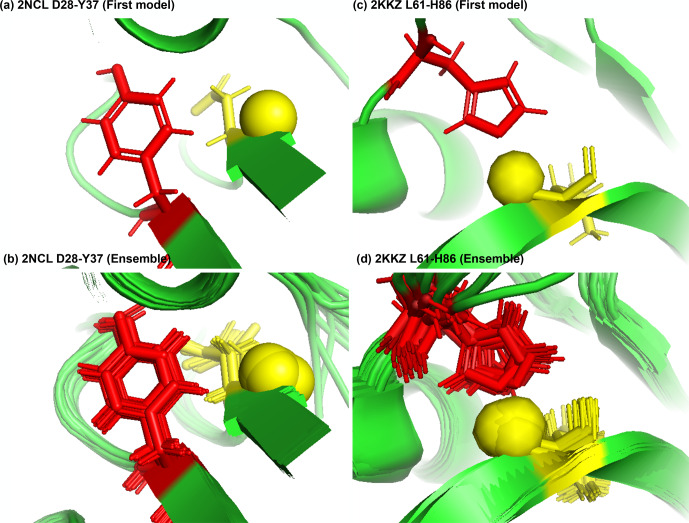
Examples of amide protons with extreme downfield shifts. **(a, b)** PDB:2NCL. The D28 amide proton is near the plane of Y37 aromatic ring ( Z = 5.21, $\delta_{\mathrm{ASP}}$ = 11.387 ppm, d = 5.62 Å, θ = 72.0 ${}^{\circ }$ , ${\bar{\delta }}_{\mathrm{ASP}}$ = 8.299 ppm, $\sigma_{\mathrm{ASP}}$ = 0.588 ppm). **(c, d)** PDB:2KKZ. The L61 amide proton forms a hydrogen bond with the sidechain nitrogen of H86 ( Z = 6.66, $\delta_{\mathrm{LEU}}$ = 12.56 ppm, d = 3.22 Å, θ = 69.7 ${}^{\circ }$ , ${\bar{\delta }}_{\mathrm{LEU}}$ = 8.217 ppm, $\sigma_{\mathrm{LEU}}$ = 0.735 ppm). The amide proton is represented as a yellow sphere, and the aromatic sidechain is shown in red.

In Fig. 6 the restrained amide–aromatic pairs are separated by the type of the aromatic residue and the number of restraints between the amide proton and the aromatic ring protons. For every aromatic type, a greater proportion of the upfield-shifted pairs have more than one restraint between them than the downfield-shifted pairs, which may indicate a hydrogen bond from the amide to the p- π electrons. This observation is consistent with the others. Finally, the prevalence of defined restrained pairs with an upfield outlier amide is quite high. From the 2529 entries considered, there were 887 such pairs, more than one in every three entries.

### 3.3 Examples

Figure 7a and b show the examples of p- π hydrogen bond in the anti-HIV lectin Oscillatoria agardhii agglutinin (PDB ID:2MWH), in which the amide chemical shifts of G93 ( z score = - 7, $\delta_{H}$ = 2.937 ppm) and G26 ( z score = - 6.43, $\delta_{H}$ = 3.38 ppm) are upfield-shifted due to the interaction of W23 and W90 respectively.

Figure 8a shows the amide proton of D28 is approximately in the plane of the Y37 aromatic ring in BOLA3 protein (PDB ID:2NCL), causing the amide chemical shift of D28( z score = 5.21, $\delta_{H}$ = 11.387 ppm) to shift downfield. Figure 8b shows an example of possible hydrogen bond between the NE2 of H86 and the amide proton of L61 in the NS1 effector domain (PDB ID:2KKZ). As a result, the L61 ( z score = 6.66, $\delta_{H}$ = 12.66 ppm) amide chemical shift is strongly downfield-shifted.

### 3.4 Bias, structure, and dynamics

Potential bias in the BMRB and PDB data likely undercounts the occurrence of aromatic hydrogen bonds. Absent assigned NOEs, the likelihood that an NMR structure will reflect a hydrogen bond to a π cloud of an aromatic ring is low because the additive force fields used to refine most NMR structures, such as X-PLOR/CNS, do not capture the favorable interaction energy. To explore the van der Waals interactions in an H-bonding geometry, we used MoSART (Hoch and Stern, 2003) to simulate ALA approaching PHE, with the amide N–H of the former exactly aligned with the ring normal of the latter. The AMBER99 force field (Wang et al., 2000) was used to compute the energy.

**Figure 9:**
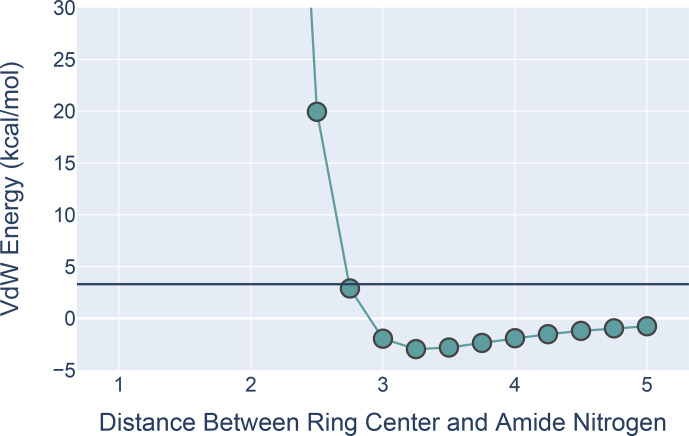
The van der Waals interaction energies for ALA approaching PHE with its amide N-H aligned with the ring normal. On the x axis is the distance from the ALA nitrogen to the PHE ring center. VdW interaction energies for each distance were calculated by subtracting the VdW energies of ALA and PHE in isolation from the energies calculated at that distance from one another. All calculations were performed in MoSART using the AMBER99 force field.

**Figure 10:**
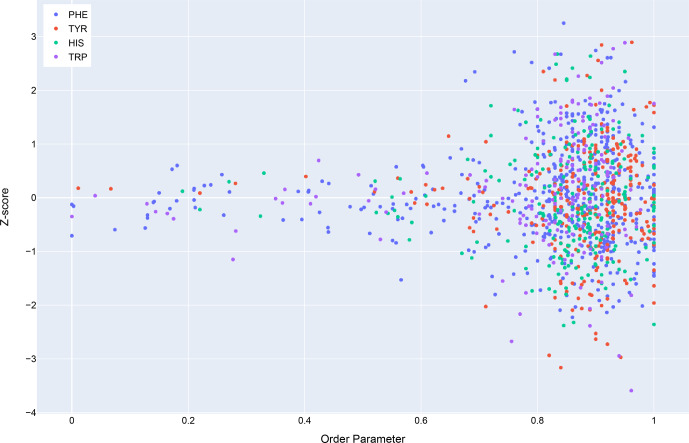
Correlation of Z scores with order parameters.

The results, shown in Fig. 9, agree with those presented by Levitt and Perutz (1988): there is a local minimum in the van der Waals (VdW) energy with the amide nitrogen 3.3 Å from the ring center. The calculations also show that the non-bonded VdW interactions do not preclude adoption of a hydrogen-bonded aromatic ring; however the well depth is so small that the VdW attraction alone is likely insufficient to yield a favorable H-bond geometry without additional restraints.

Lack of assignments are not evidence of the absence of an NOE. Missing assignments (for example, 6280 out of 8111 outlying amide proton shifts ( |Z|>2 ) do not have assigned NOEs to an aromatic ring) would also lead to an undercount. Possible bias in BMRB notwithstanding, such as missing assignments not uniformly distributed, trends in shifts and NOE restraints for different amino acid types that mirror one another provide a form of cross-validation and suggest that the shift outliers are not simply the result of being buried in the protein and thus easier to assign. Bias in PDB NMR structures could reflect current practice in structure refinement, which is dominated by restrained molecular mechanics simulations using empirical force fields augmented with experimental restraint potentials. The forms of these restraint potentials can introduce bias (Hoch and Stern, 2005), and the additive potentials that are used do not explicitly model p- π hydrogen bonds. Absent NOE or ring current restraints, NMR structures are likely to under-represent aromatic hydrogen bonds.

**Figure 11:**
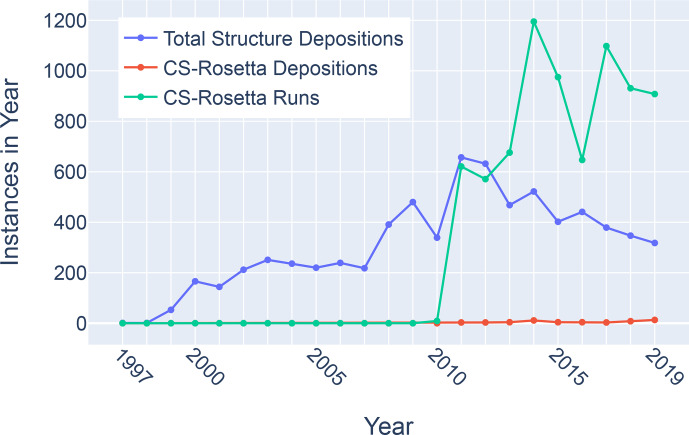
Trends in total BMRB structure depositions (blue), runs executed using the BMRB CS-Rosetta server (green), and depositions citing CS-Rosetta (red).

**Figure 12:**
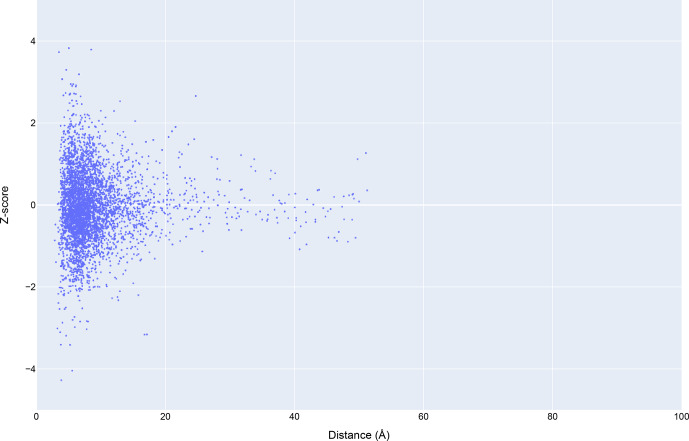
The distribution of amide chemical shifts for depositions citing CS-Rosetta as a function of distance from the center of the nearest ring (compare Fig. 3).

In general, dynamics and disorder render chemical shifts toward their random-coil or median values (Dass et al., 2020; Nielsen and Mulder, 2020). The correlation between secondary shift and order parameters is sufficiently strong that it has been used to predict order parameters from chemical shifts (Fig. 10) (Berjanskii and Wishart, 2005). Ring current effects in particular are diminished by fluctuations about the $\chi_{2}$ torsion angle (Hoch et al., 1982). Hydrogen bonds involving aromatic rings should diminish these torsional fluctuations and should find correlates in sidechain relaxation properties for aromatic residues. Solution NMR structures in general tend to be more flexible than crystal structures (Fowler et al., 2020), and inclusion of hydrogen bonding interactions between amide groups and aromatic rings could reduce the flexibility and potentially improve the accuracy of NMR structures.

Although chemical shifts have been used to refine protein NMR structures (Shen et al., 2009; Berjanskii et al., 2015; Cavalli et al., 2007), for the most part these approaches leverage the influence of backbone torsion angles on chemical shifts and do not consider the influence of nearby sidechains. Despite evidence that chemical shift refinement software is being used more frequently, the pace of chemical shift-refined structure depositions remains low (Fig. 11).

Filtering the data plotted in Fig. 3 to include only structures that reference CS-Rosetta (Fig. 12) does not alter the overall distributions. A challenge confronting a deeper understanding of these effects is that the available metadata in BMRB do not articulate workflows (for example, whether CS-Rosetta is used to generate initial trial structures or as a final refinement step), nor does it indicate when ring current shift restraints were utilized.

## 4 Concluding remarks

Ring current shifts have a long history of providing structural insights from NMR studies of globular proteins (Perkins and Dwek, 1980), especially for methyl groups, whose secondary shifts tend to be dominated by ring current shifts. Early studies were largely anecdotal, focusing on individual proteins or small surveys. While relatively dynamic aromatic rings (for example Tyr and Phe rings that undergo ring flips on the fast exchange timescale) and disorder diminish the influence of ring current effects on secondary shifts (Hoch et al., 1982), the accumulation of data in BMRB for folded proteins has provided a wealth of amide chemical shifts exhibiting large secondary chemical shifts. Federation of BMRB chemical shift data with structural data from PDB confirms the strong correlation between proximity to an aromatic ring and extreme secondary shifts. Markedly different secondary shift trends for different aromatic residue types suggest promising avenues for improving protein structure determination by NMR. Though chemical shift refinement has been repeatedly demonstrated (Perilla et al., 2017), it has not yet been widely adopted.

The extreme outlier amide chemical shifts and corroborating NOE effects examined here provide strong evidence of the widespread existence of amide–aromatic hydrogen bonds, but they are not fully conclusive. Nonetheless potential for under-representation in the BMRB data exists because of incomplete assignments. Relaxation studies on ring dynamics, contrasting rings where evidence suggests the presence of hydrogen bonding with rings lacking such evidence, could provide additional corroboration. Molecular mechanics simulations and structure refinement using polarizable force fields could reveal additional aromatic hydrogen bonds and restricted ring dynamics in folded proteins. We have initiated investigations along some of these lines.

More broadly, this preliminary investigation highlights the potential for unlocking latent knowledge hidden in BMRB, PDB, and other biological databases. The challenges posed include curation and validation of the data repositories and federation of data between repositories. Robust and efficient solutions to these challenges are needed in order to realize the full promise of emerging methods in machine learning (Hoch, 2019).

## Supplement

10.5194/mr-2-765-2021-supplement The supplement related to this article is available online at: https://doi.org/10.5194/mr-2-765-2021-supplement.

## Data Availability

Curated datasets generated for this study are available on BMRbig (https://doi.org/10.13018/bmrbig29, Baskaran and Wilburn, 2021).

## Code availability

Source code developed for this project by Kumaran Baskara and Colin W. Wilburn is available on GitHub (https://github.com/uwbmrb/rcs, Wilburn, and Baskaran, 2021​​​​​​​) and on the NMRbox platform (https://nmrbox.org/, last access: 27 August 2021, path: /public/rcs).
