# Supplementary material for: Anomalous amide proton chemical shifts as signatures of hydrogen bonding to aromatic sidechains
Source: Magn Reson (Gott). 2021 Oct 25;2(2):765–75. doi: 10.5194/mr-2-765-2021 (PMC10539802; doi:10.5194/mr-2-765-2021)
Supplement: The supplement related to this article is available online at: https://doi.org/10.5194/mr-2-765-2021-supplement. [file mr-2-765-supplement.pdf]

Supplement of Magn. Reson., 2, 765–775, 2021
https://doi.org/10.5194/mr-2-765-2021-supplement
© Author(s) 2021. CC BY 4.0 License.

Open Access
MAGNETIC RESONANCE

Supplement of

Anomalous amide proton chemical shifts as signatures of hydrogen bonding to aromatic sidechains

Kumaran Baskaran et al.

Correspondence to: Jeffrey C. Hoch (hoch@uchc.edu)

The copyright of individual parts of the supplement might differ from the article licence.

**Reason for Exclusion**	**# Excluded**	**Examples (BMRB ID, PDB ID)**
DNA/RNA entries, entries with ligands, oligomers and protein complexes	1767	(50411, 7JK8)
Severe residue index mismatch between PDB and BMRB	1199	(50842, 7MC3)
Restraint file not in ReBoxitory	716	(36368, 7CQ1)
No distance restraints in restraints file	190	(50264, 6Z0H)
No aromatic residues in sequence	152	(50202, 6YFY)
>3500 distance restraints *	151	(36176, 5ZMR)
Restrained amide-aromatic pairs > 8Å apart *	136	(36008, 5GHD)
Unable to match residues from structure to restraints	99	(36097, 5XR1)
mmCIF file not in ReBoxitory	18	(50886, 7MLL)
STR file not in ReBoxitory	7	(36326, 6M6K)
Empty restraint file	3	(25068, 2MRC)
Misformatted restraint file	3	(4540, 1NLA)
Miscellaneous	7	(2NBN, 25984), (2M7V, 19214), (2LK9, 17985), (2KSI, 16665), (1SF0, 6187), (1RFH, 6059), (1P9F, 5864)

Table S1: Reasons for the exclusion of BMRB/PDB entries from the analysis. Most reasons make analysis difficult (e.g. index mismatches) or impossible (e.g. no restraint file). Reasons with a * were implemented to maintain quality. An entry having > 3500 distance restraints or restraints between amide-aromatic pairs that were > 8 Å apart was in some cases indicative of deeper problems with the restraint assignments, such as restraints being assigned between all atoms.
